# Characterization and application of in situ curcumin/ZNP hydrogels for periodontitis treatment

**DOI:** 10.1186/s12903-024-04054-7

**Published:** 2024-03-28

**Authors:** Chengcheng Liu, Ying Chen, Huimin Bai, Yulong Niu, Yafei Wu

**Affiliations:** 1 State Key Laboratory of Oral Diseases & National Center for Stomatology & National Clinical Research Center for Oral Diseases & Department of Periodontics, West China Hospital of Stomatology, Sichuan University, No.14, 3rd Section of Renmin South Road, Chengdu, 610041 Sichuan China; 2 College of Life Science, Sichuan University, No.24, 1st South Section, Yihuan Road, Chengdu, 610065 Sichuan China

**Keywords:** Curcumin, Zinc oxide nanoparticles, *P. Gingivalis*, Bone loss

## Abstract

**Background:**

Periodontitis is a chronic inflammatory disease that occurs in tooth-supporting tissues. Controlling inflammation and alleviating periodontal tissue destruction are key factors in periodontal therapy. This study aimed to develop an in situ curcumin/zinc oxide (Cur/ZNP) hydrogel and investigate its characteristics and effectiveness in the treatment of periodontitis.

**Methods:**

Antibacterial activity and cytotoxicity assays were performed in vitro. To evaluate the effect of the in situ Cur/ZNP hydrogel on periodontitis in vivo, an experimental periodontitis model was established in Sprague‒Dawley rats via silk ligature and inoculation of the maxillary first molar with *Porphyromonas gingivalis*. After one month of in situ treatment with the hydrogel, we examined the transcriptional responses of the gingiva to the Cur/ZNP hydrogel treatment and detected the alveolar bone level as well as the expression of osteocalcin (OCN) and osteoprotegerin (OPG) in the periodontal tissues of the rats.

**Results:**

Cur/ZNPs had synergistic inhibitory effects on *P. gingivalis* and good biocompatibility. RNA sequencing of the gingiva showed that immune effector process-related genes were significantly induced by experimental periodontitis. Carcinoembryonic antigen-related cell adhesion molecule 1 (Ceacam1), which is involved in the negative regulation of bone resorption, was differentially regulated by the Cur/ZNP hydrogel but not by the Cur hydrogel or ZNP hydrogel. The Cur/ZNP hydrogel also had a stronger protective effect on alveolar bone resorption than both the Cur hydrogel and the ZNP hydrogel.

**Conclusion:**

The Cur/ZNP hydrogel effectively inhibited periodontal pathogenic bacteria and alleviated alveolar bone destruction while exhibiting favorable biocompatibility.

**Supplementary Information:**

The online version contains supplementary material available at 10.1186/s12903-024-04054-7.

## Background

The best method for the prevention and treatment of periodontitis is scaling and root planing (SRP), combined with effective self-performed supragingival plaque control measures [1]. SRP is considered the “gold standard” for periodontal treatment because it can be used to remove calculus and to effectively create a biologically acceptable root surface for long junctional epithelial attachment [2]. However, SRP has several drawbacks: (1) it cannot completely remove bacterial and toxin residues, and (2) it causes discomfort and bleeding [3]. Common adjuvant treatment methods, such as systemic or local application of antibiotics, also have certain deficits, including resistance to bacteria, adverse drug reactions, and drug cross-reactions [1]. Therefore, identifying new drugs and methods to assist SRP in the treatment of periodontitis is important.

Curcumin (Cur) is a hydrophobic yellow polyphenolic compound with various biological activities (e.g., antibacterial, anti-inflammatory, antioxidant, and antitumor) [4, 5]. Oral administration of Cur can relieve bone absorption caused by periodontitis and reduce the expression of interleukin-4 (IL-4) and tumor necrosis factor-α (TNF-α) in the gingiva [6, 7]. Moreover, SRP with 2% Cur gel for one month significantly reduced the probing depth (PD), bleeding index (BI), and plaque index (PLI) of patients with periodontitis [8]. Although Cur has excellent antioxidant and anti-inflammatory effects, there are urgent issues that limit its bioavailability and clinical application, such as poor solubility in water (approximately 11 ng/mL), low oral absorption (approximately 1%), rapid systemic metabolism to complexes (e.g., glucuronide and sulfate), and fast photodegradation by light [5]. Thus, different strategies for incorporating Cur in drug delivery systems, including hydrogels, nanofibers, lyotropic liquid crystal systems, and micelles, have been investigated [9–11]. Hydrogels are three-dimensional polymer network structures that can enhance the bioavailability of Cur [12]. Methylcellulose is an attractive material for the production of hydrogels. For instance, nanocellulose-reinforced chitosan hydrogels could enhance the bioavailability of Cur for absorption from the stomach and upper intestinal tract [13]. Emerging evidence has also indicated that zinc oxide nanoparticles (ZNPs) have great potential for improving the delivery and bioactivity of Cur [14]. ZNPs have also been shown to promote the expression of osteogenesis-related genes in fibroblasts and inhibit the growth of periodontal pathogens such as *P. gingivalis* and *Fusobacterium nucleatum* [15, 16]. Therefore, the synthesis of Cur/ZNP hydrogels is a promising strategy for improving the bioavailability of Cur and enhancing its role in the treatment of periodontitis. In this study, a Cur/ZNP hydrogel was synthesized to evaluate its properties, biocompatibility, and inhibitory effect on *P. gingivalis* in vitro. The effect of Cur/ZNP hydrogel injection in the periodontal pocket on periodontal destruction was also investigated using a model of experimental periodontitis.

## Methods

### Preparation of hydrogels and drug release

Methylcellulose (Sigma‒Aldrich, LLC) was slowly added to deionized water at 70 °C, after which the mixture was oscillated to form a 14% methylcellulose solution; the mixture was subsequently stored at 4 °C overnight to ensure complete wetting and removal of entrapped air bubbles, thus forming the hydrogel. A Cur hydrogel was prepared by weighing pure Cur powder with a microbalance, slowly adding Cur to the preprepared methylcellulose hydrogel, and stirring in an ice bath. Cur methylcellulose hydrogels at 1 mg/mL, 2 mg/mL, and 3 mg/mL were prepared, and then all the samples were transferred to amber bottles and stored in a freezer. A ZNP methylcellulose hydrogel was prepared by adding 25 mg, 50 mg, or 75 mg of a ZNP dispersion (40 wt %) into 10 mL of the hydrogel prepared in advance in an ice bath. The mixture was stirred evenly to prepare 1 mg/mL, 2 mg/mL, and 3 mg/mL ZNP hydrogels. A Cur/ZNP hydrogel was prepared by adding 10 mg of pure curcumin powder and 25 mg of a ZNP dispersion (40 wt %) to a 10 mL hydrogel and stirring it evenly to obtain a Cur/ZNP hydrogel at 2 mg/mL. Cur/ZNP hydrogels at 1 mg/mL and 3 mg/mL were prepared in the same way. The Cur hydrogel was yellow and translucent, the ZNP hydrogel was white and translucent, the Cur/ZNP hydrogel was yellow, and the color was darker than that of the Cur hydrogel. The injectability and fluidity of the hydrogels were evaluated at different temperatures. To test drug release in vitro, Cur hydrogel (2 mL) and ZNP hydrogel (2 mL) were placed in centrifuge tubes and slowly added to 20 mL of phosphate-buffered saline (PBS) release medium containing 0.5% Tween-80. The release system was placed in a horizontal shaker at 37 °C and 100 rpm. The upper release medium (1 mL) was removed at a specific time, and the OD_431nm_ and OD_268nm_ were determined; then, 1 mL of fresh release medium was added. During the experiment, no flocs or precipitates appeared in the release medium. Three replicates of each hydrogel were performed. The drug concentration at each time point was calculated according to the standard curve (Additional Fig. 1), and the cumulative percentage release of Cur and ZNPs was calculated according to the following formula (${\text{E}}_{R}$) [17]:

$${\text{E}}_{R}=\frac{{V}_{e}{\sum }_{1}^{n-1}{C}_{i}+{V}_{0}{C}_{n}}{{m}_{i}}\times 100$$

where ${m}_{i}$is the initial dose of drug loaded in the hydrogel (mg), ${V}_{0}$ is the total volume of release medium (${V}_{0}$ = 20 mL), ${V}_{e}$ is the volume of replaced release medium (${V}_{e}$ = 1 mL), and ${C}_{n}$ is the drug concentration of the n-th sample (${V}_{e}$ = 1 mL).

### Cells and cell culture

*P. gingivalis* ATCC 33,277 was cultured anaerobically at 37 °C in Trypticase soy broth supplemented with yeast extract (1 mg/ml), hemin (5 μg/mL) and menadione (1 μg/mL). Human oral keratinocyte-1 (HOK-1) cells were cultured in oral keratinocyte medium (ScienCell Research Laboratories, Inc.) in an incubator at 37 °C with 5% CO_2_.

### Antibacterial activity evaluation

Leaching solutions of hydrogels at different concentrations (1, 2, or 3 mg/mL) were prepared [18]. *P. gingivalis* (20 μL 10^9^ CFU/mL) and 200 μL of leaching solution were incubated anaerobically at 37 °C in a 96-well plate for 24 h, after which the OD_600nm_ was measured. To determine the influence of the hydrogels on the growth of *P. gingivalis*, 20 μL of 10^9^ CFU/mL *P. gingivalis* solution was incubated with 2 mg/mL hydrogel. The absorbance at OD_600nm_ was measured at 0, 12, 24, and 48 h.

### Scanning electron microscopy (SEM) and confocal laser scanning microscopy

*P. gingivalis* treated with each hydrogel was fixed on cover glass slides at 4 °C for 2 h in 2.5% glutaraldehyde, followed by washing with PBS buffer and gradient dehydration with alcohol and anhydrous ethanol. After sputtering with a layer of gold, the morphology of *P. gingivalis* was observed using a scanning electron microscope (Hitachi S-3400). A laser scanning microscope (Leica) was used to detect exopolysaccharides (EPSs) in *P. gingivalis*. EPSs were labeled with the tetramethylrhodamine isothiocyanate-labeled lectin concanavalin A from *Canavalia ensiformis* (TRITC-Con A; Molecular Probes, Inc., USA), and *P. gingivalis* was labeled with fluorescein isothiocyanate (FITC) [19].

### Cell cytotoxicity assay

The cytotoxicity of Cur and ZNPs on HOK-1 cells was examined using a CCK-8 assay (APExBIO Technology LLC). Briefly, 10^4^ cells were seeded in each well of a 96-well plate and cultured at 37 °C for 24 h in 5% CO_2_. The medium was replaced with 100 μL of leaching solution, and the mixture was cultured for 24 h. The medium was subsequently replaced with medium containing 10% CCK-8, the mixture was incubated at 37 °C for 1 h, and the OD_450nm_ was detected.

### Animal study design

All animal procedures were approved by the Ethics Committee of the State Key Laboratory of Oral Diseases, West China Hospital of Stomatology, Sichuan University (WCHSIRB-D-2020-438); the protocols ensured humane practices. The animal experimental study was conducted using eight-week-old male Sprague‒Dawley rats (weight, 250–300 g) purchased from Chengdu Dashuo Experimental Animal Co., Ltd. (license no. 510,109,000,176,387). The rats were randomly divided into five groups (*n* = 5 per group): (i) healthy controls (sham), (ii) experimental periodontitis (EP), (iii) experimental periodontitis treated with Cur hydrogel (Cur gel), (iv) experimental periodontitis treated with ZNP hydrogel (ZNP gel), and (v) experimental periodontitis treated with Cur/ZNP hydrogel (Cur/ZNP gel). According to the procedure and method in Additional Fig. 2, the EP was established using ligatures with 4–0 silk thread around the bilateral maxillary first molars. *P. gingivalis ATCC 33,277* (10^7^ CFU/mL) was smeared onto silk twice a week. One month after ligature placement, rats in the EP + Cur group were treated with Cur hydrogel (Dalian Meilun Biotech Co., Ltd.), those in the EP + ZNP group were treated with ZNP hydrogel (Shanghai Aladdin Biochemical Technology Co., Ltd.), and those in the EP + Cur/ZNP group were treated with Cur/ZNP hydrogel. To do so, 200 μL of each hydrogel (2 mg/mL) was injected into the periodontal pocket of the bilateral maxillary first molar once a week for one month with a blunt syringe. Hydrogels were used as a control for the sham group. Rats were excluded if rapid weight loss and mental or behavioral burnout occurred. According to the distribution of different stages in the experiment, there were corresponding records on the label outside the cage. Outcome evaluation and data analysis were carried out based on random cage units by one investigator who was blinded to the group assignments in the experiments. One month after hydrogel treatment, there was no significant difference in the body weight of the rats between the groups (Additional Table 1). All rats were anesthetized, and blood was drawn by cardiac puncture and euthanized by CO_2_ inhalation. The maxillary jaws and gingiva were then harvested.

### Periodontal destruction analyses

Maxillary jaw samples were decalcified in 10% ethylenediaminetetraacetic acid (EDTA) solution for two months before being sequentially dehydrated in ethyl alcohol at different concentrations. Finally, the tissues were processed into paraffin sections for hematoxylin and eosin (H&E) staining to facilitate the observation of the alveolar bone level (ABL) under a light microscope (DM 500; Leica). A high-resolution micro-CT system (μCT 50; SCANCO Medical AG) was used to scan the maxillary jaw samples [20].

### Immunohistochemical (IHC) staining and enzyme-linked immunosorbent assay (ELISA)

IHC staining was performed to highlight osteocalcin (OCN)-positive osteoblasts (ColofulGene Bio, Inc.) and to detect osteoprotegerin (OPG) expression (ColofulGene Bio, Inc.). The number of positive cells per square millimeter of bone marrow was observed under a light microscope and counted using Image-Pro Plus 6.0 software (Media Cybernetics, Inc.). Epicanthus venous blood was centrifuged (10 min, 2432 × *g*) at 4 °C to obtain serum. The serum levels of OCN were measured using corresponding ELISA kits (Thermo Fisher Scientific, Inc.).

### RNAseq and quantitative (q) reverse transcription (RT)-PCR

Total RNA was extracted using TRIzol® reagent (Thermo Fisher Scientific, Inc.). The TruSeq Stranded total RNA with the RiboZero Plus kit (IIlumina, San Diego, CA) was used to obtain a sequencing library from 1 μg of total RNA. Eukaryotic mRNA sequencing was performed using an Illumina NovaSeq 6000 by Shanghai Majorbio Biopharm Technology Co., Ltd. Raw gene counts with a minimum of two counts per million in at least one sample were used for downstream analyses. Differentially expressed genes were determined using the DESeq2 R package [21]. GO enrichment analysis was conducted using the GO seq package, and the results were visualized using the clusterProfiler package [22, 23]. The GO gene network and PPI network were visualized using Cytoscape [24]. All sequencing reads were deposited in the Gene Expression Omnibus (GEO) database under the accession number GSE185147. qPCR was performed using TaqMan Real-Time PCR Master Mix (Thermo Fisher Scientific, Inc.) and TaqMan gene expression assays (Thermo Fisher Scientific, Inc., Assay IDs Rn01476373_g1 and Rn01775763_g1). The transcription of *Ceacam1* in the gingiva of rats was evaluated using the 2^−ΔΔCT^ method, with *Gapdh serving* as an internal reference.

### Statistical analysis

Analysis of variance with Tukey’s multiple comparison test was conducted using GraphPad Prism V8.0.0 (GraphPad Software, Inc.), and a *P* value < 0.05 indicated statistical significance. The values are expressed as the mean ± standard deviation.

## Results

### The structure, cumulative release rate, and biocompatibility of the Cur/ZNP hydrogels

**Fig. 1:**
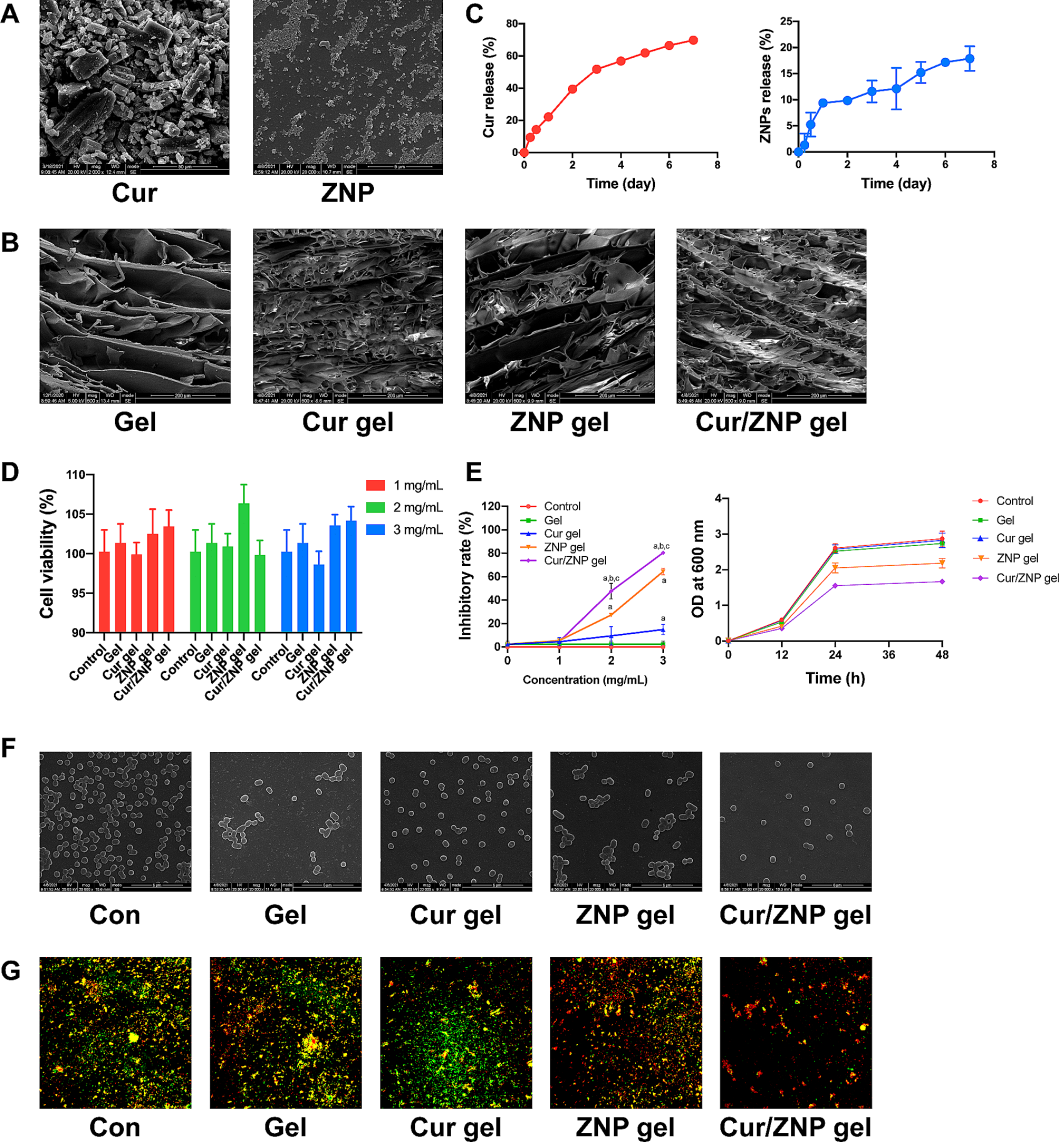
The structure of the Cur/ZNP hydrogel and its effect on *P. gingivalis*. (**A**). Representative SEM images showing the microstructures of Cur and ZNPs. (**B**). Representative SEM images showing the microstructure of the hydrogels. (**C**). Cumulative release rates of Cur and ZNPs. (**D**). The activity of HOK-1 cells after treatment with hydrogels for 24 h. (**E**). Effects of different concentrations of Cur/ZNP hydrogels on *P. gingivalis*. (**F**). Representative SEM images of *P. gingivalis* after treatment with hydrogels for 24 h. (**G**). Representative confocal laser scanning microscopy images of EPSs (red) produced by *P. gingivalis* (green). The error bars indicate the means ± standard deviations. SEM, scanning electron microscopy; Cur, curcumin; ZNP, zinc oxide nanoparticle; EPS, exopolysaccharide; HOK-1, human oral keratinocyte-1

Cur is approximately 10 μm in size, and ZNPs are particles with a diameter of approximately 10 nm (Fig. 1A). The hydrogels had a porous three-dimensional network structure with pores of approximately 10–100 μm. The loading of drugs had no effect on the microstructure of the hydrogels (Fig. 1B). The hydrogels exhibited good injectability and fluidity at 4 °C. At 20 °C, the hydrogel was injectable and fluid, but its injectivity and fluidity were weaker than those at 4 °C. At 37 °C, the hydrogel has almost no fluidity, but it can be injected. (Additional Table 2). The data obtained from in vitro drug release experiments were plotted as the cumulative percentage of drug release versus time (Fig. 1C). The cumulative release rate of Cur was approximately 50% during the first three days, at which point it gradually stabilized. After one week, the cumulative release rate reached 70% (Fig. 1C). The release rate of the ZNPs was rapid on the first day, and the cumulative release rate reached approximately 20% after one week (Fig. 1C). The CCK-8 assay results indicated that all the hydrogels (1–3 mg/mL) had no significant cytotoxic effects on HOK-1 cells (Fig. 1D).

The ***Cur/ZNP hydrogel inhibited the growth and EPS production of P. gingivalis***.

The Cur, ZNP, and Cur/ZNP hydrogels inhibited the growth of *P. gingivalis* in a concentration-dependent manner at 24 h (Fig. 1E). The Cur/ZNP hydrogel had a stronger antibacterial effect than the ZNP hydrogel alone, and the antibacterial rate of the Cur/ZNP hydrogel at 2 mg/mL was 50%. Within 48 h, the 2 mg/mL ZNP hydrogel and Cur/ZNP hydrogel had a sustained inhibitory effect on the growth of *P. gingivalis*, and the Cur/ZNP hydrogel had a stronger inhibitory effect than the ZNP hydrogel alone (Fig. 1E). The morphology of *P. gingivalis* was not influenced by the hydrogel (Fig. 1F). EPS production by *P. gingivalis* decreased after treatment with the 2 mg/mL Cur hydrogel or Cur/ZNP hydrogel (Fig. 1G).

### The Cur/ZNP hydrogel modulated the bone resorption signaling pathway in vivo

**Fig. 2:**
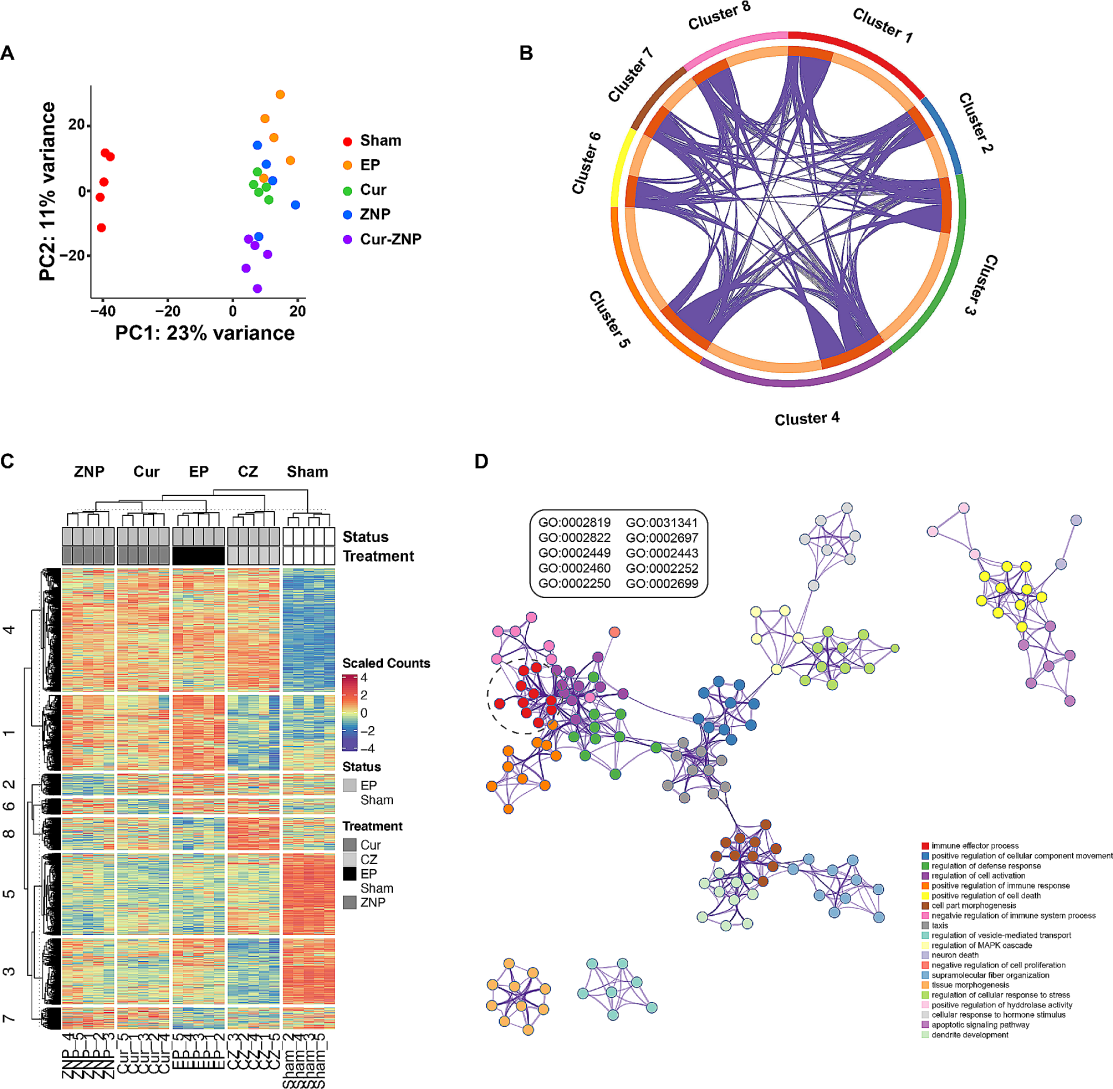
Transcriptional response pattern of the gingiva of rats after treatment with Cur/ZNP hydrogels. (**A**). Transcriptional patterns of the gingiva of rats treated with different hydrogels showing clear divergence as determined by principal component analysis. (**B**) Cluster and (**C**) heatmap of differentially expressed genes (DEGs) obtained by pairwise comparison. The scaled counts of the transcripts are marked on the right. (**D**) Changes in GO terms (adjusted *P* < 0.05) between the sham and EP groups. The colors of the circles correspond to the functional terms on the right. The DEGs are significantly enriched in the immune effector process GO terms, as shown in the box. Cur, curcumin; ZNP, zinc oxide nanoparticle; GO, Gene Ontology; EP, experimental periodontitis

**Fig. 3:**
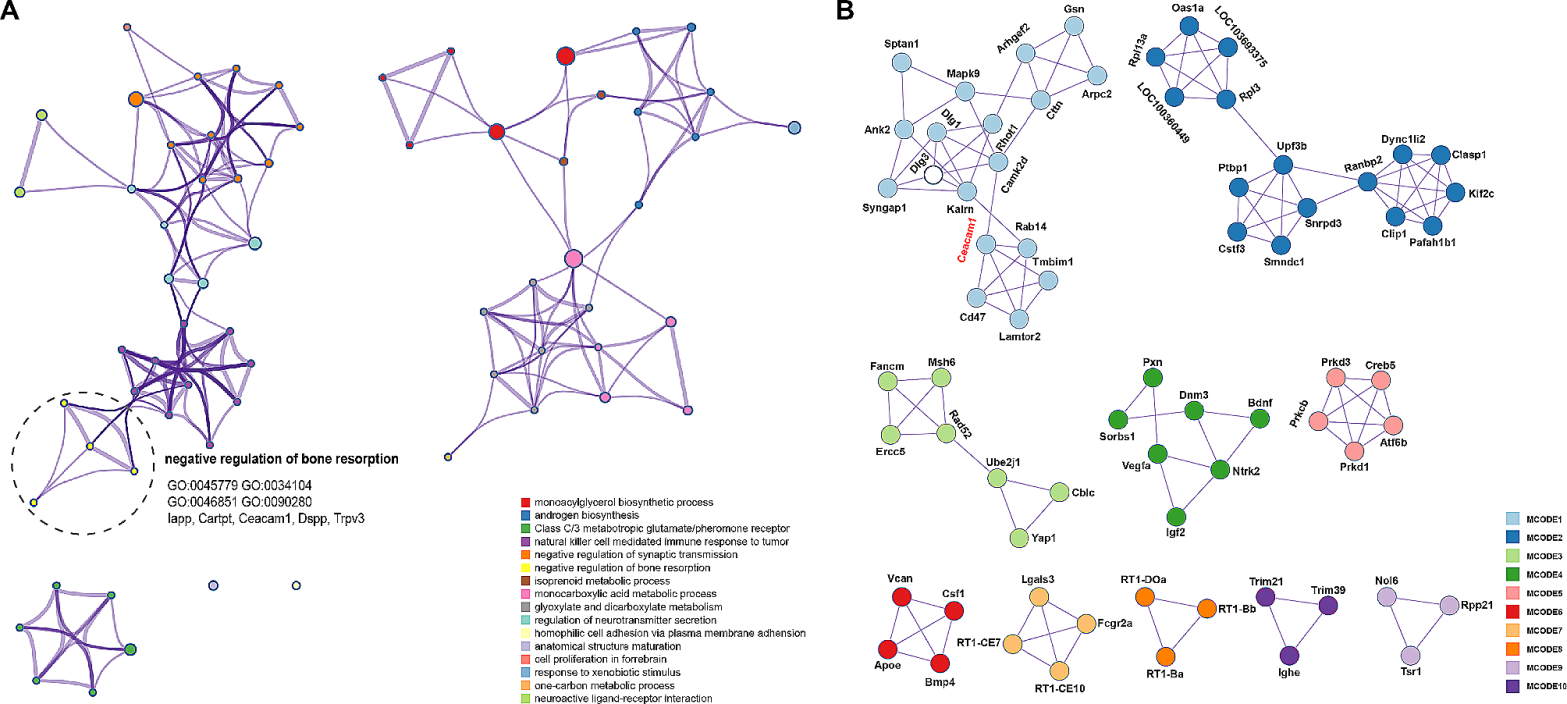
Enriched GO terms and PPI networks of DEGs induced by the Cur/ZNP hydrogel. (**A**). The GO terms (adjusted *P* < 0.05) were related to the differences between the Cur/ZNP gel group and the Cur gel or ZNP gel single treatment group. The colors of the circles correspond to the functional terms on the right. (**B**) The PPI network of DEGs between the Cur/ZNP gel group and the Cur gel or ZNP gel single treatment group. GO, Gene Ontology; PPI, protein‒protein interaction; DEG, differentially expressed gene; Cur, curcumin; ZNP, zinc oxide nanoparticle

To further explore the effect of the Cur/ZNP hydrogel on the gingiva in vivo, we measured the transcriptional profile of the gingiva of the rats. The distance of the transcriptomes corresponding to the five groups was determined by principal component analysis (PCA) (Fig. 2A). The groups are marked with distinct colors, and the first principal component (PC1), which had the largest variance (23%), separated the sham and EP groups and treatment groups most. Interestingly, the EP group was further separated by the second principal component (PC2), for which the variance was 11%. *K*-means clustering of the differentially expressed genes (DEGs) involved in the different treatments revealed that eight large clusters were induced or suppressed (Fig. 2B and C). Gene Ontology (GO) analysis revealed that DEGs between the sham and EP groups were significantly enriched in the following GO terms: immune effector process, positive regulation of cellular component movement, and regulation of defense response (Fig. 2D). Importantly, compared with those in the Cur hydrogel and ZNP hydrogel groups, the expression of the genes in the Cur/ZNP hydrogel group was significantly greater according to the GO term, indicating that the combination treatment negatively regulates bone resorption (Fig. 3A). The expression of a member of the immunoglobulin superfamily, carcinoembryonic antigen-related cell adhesion molecule 1 (Ceacam1), was upregulated in the gingiva of rats in the EP group (FC = 2.1). Additionally, Cur/ZNP treatment inhibited the expression of Ceacam1 compared with that in the Cur hydrogel (FC = 2.4) and ZNP hydrogel groups (FC = 1.8) (Fig. 3A and Additional Fig. 3). Protein‒protein interaction (PPI) network analysis via topological and functional module identification showed that Ceacam may interact with the surface glycoprotein CD47 (Fig. 3B).

### The Cur/ZNP hydrogel attenuates alveolar bone loss in rats with periodontitis

**Fig. 4:**
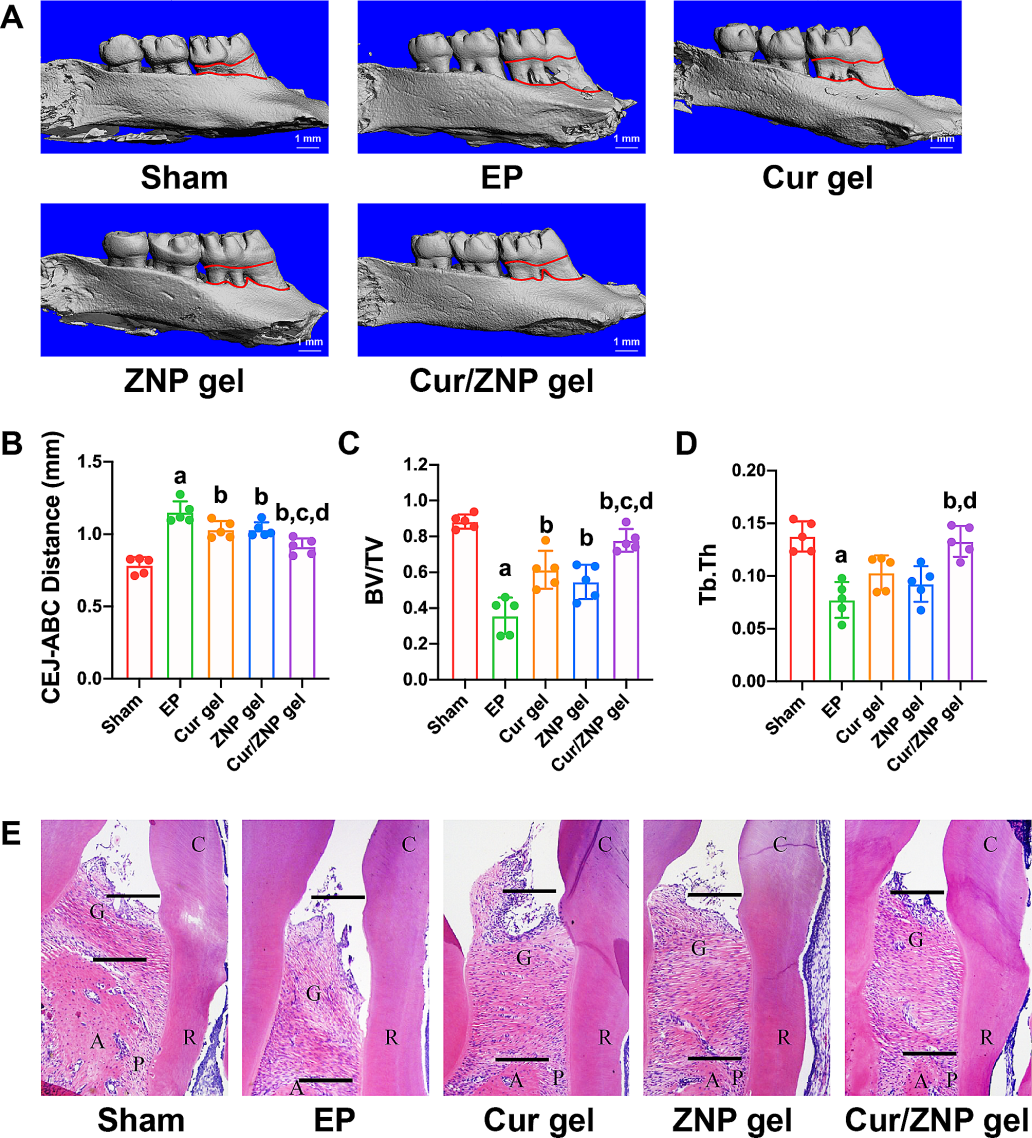
Role of the Cur/ZNP hydrogel in ameliorating alveolar bone loss in rats with experimental periodontitis. (**A**). Micro-CT reconstruction revealed alveolar bone loss in the maxillary first molars. (**B**). Quantitative analysis of alveolar bone loss in the maxillary first molar at six sites. The sagittal plane parallel to the long axis of the teeth was chosen to measure the distance between the cemento-enamel junction and the alveolar bone crest (CEJ-ABC) to assess the ABL at the interproximal site. (**C**). Quantitative analysis of the bone volume/total volume (BV/TV) of the region of interest (ROI) in the first molar beneath the furcation area. A 500 × 300 μm^2^ region apical to the root furcation was chosen as the ROI, and a three-dimensional reconstruction model was used to analyze the bone parameters in the ROI. (**D**). Quantitative analysis of the trabecular thickness (Tb.Th) of the ROI in the first molar beneath the furcation area. (**E**). Hematoxylin and eosin staining of distal periodontal tissue of the maxillary first molar of rats. The data are presented as the mean ± standard deviation (*n* = 5 rats per group). A, alveolar bone; C, crown; G, gingiva; P, periodontal membrane; R, root; CEJ-ABC, cemento-enamel junction and alveolar bone crest. a, Compared with the sham group; ^*^P < 0.05; b, compared with the EP group, ^*^P < 0.05; c, compared with the Cur gel group, ^*^P < 0.05; d, compared with the ZNP gel group, ^*^P < 0.05. Cur, curcumin; ZNP, zinc oxide nanoparticle; EP, experimental periodontitis

Alveolar bone resorption, which is the typical feature of periodontitis, was induced via ligature and *P. gingivalis* inoculation of the maxillary first molar (Fig. 4A-E). The CEJ-ABC distance increased significantly in the EP group compared to the sham group (Fig. 4B). The bone volume fraction (BV/TV) and trabecular thickness (Tb.Th) were significantly lower in the EP rats than in the sham rats (Fig. 4C and D). Histological analysis revealed obvious periodontal pockets and a decreased alveolar bone height in the EP group (Fig. 4E). Treatment with the Cur hydrogel, ZNP hydrogel, or Cur/ZNP hydrogel alleviated the alveolar bone loss induced by EP (Fig. 4A-E). Notably, Cur/ZNPs had a stronger protective effect on alveolar bone resorption than both Cur and ZNPs, which was supported by a shorter CEJ-ABC distance (Fig. 4B) and a greater BV/TV and Tb.Th (Fig. 4C and D).

**Fig. 5:**
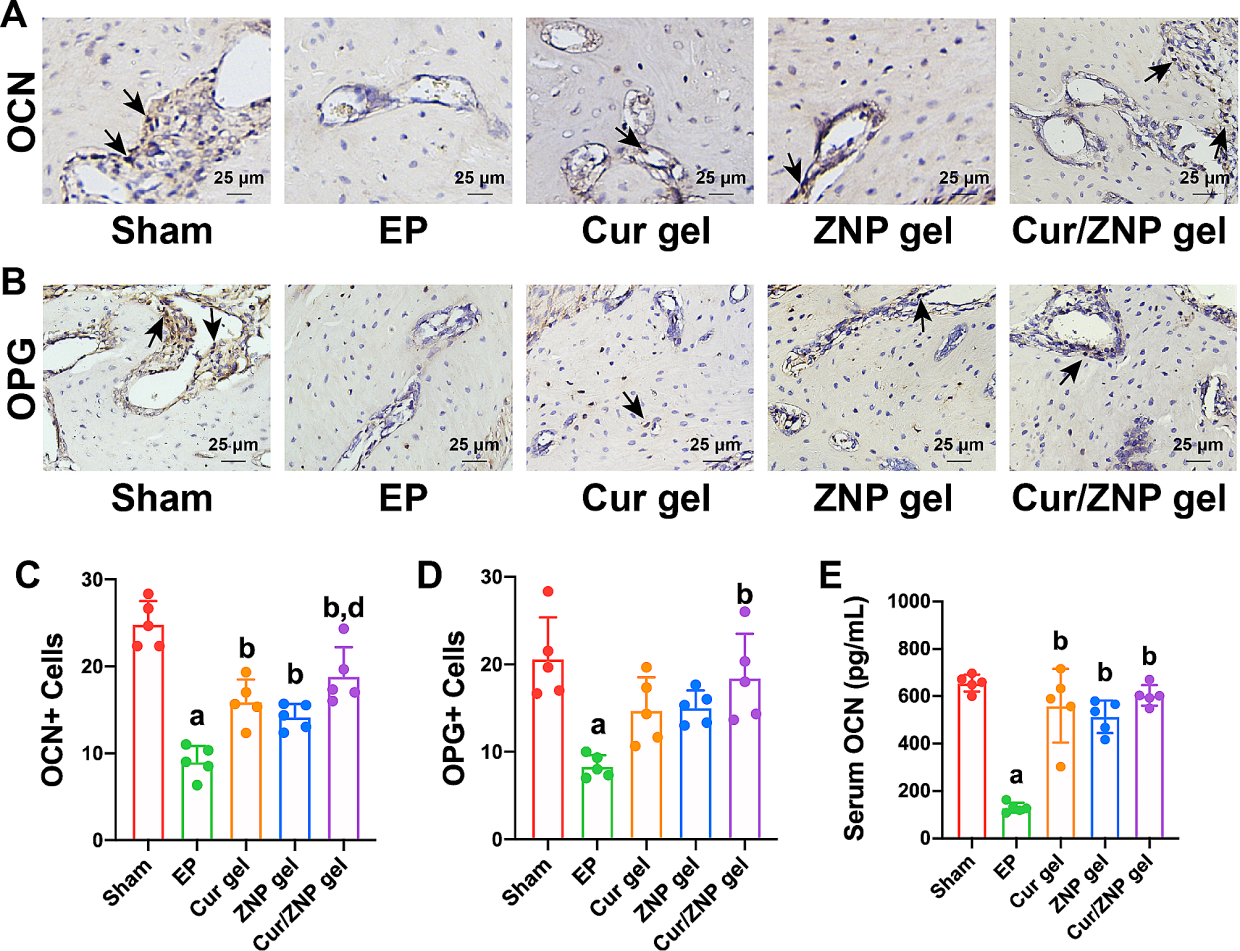
Effect of the Cur/ZNP hydrogel on bone metabolism in rats with experimental periodontitis. (**A**). The effect of the Cur/ZNP hydrogel on OCN in the alveolar bone of rats was determined by immunohistochemical staining. (**B**). The effect of the Cur/ZNP hydrogel on OPG in the alveolar bone of rats was determined by immunohistochemical staining. The black arrows indicate the staining of positive cells. Quantitative analysis of OCN^+^ cells (**C**) and OPG^+^ cells (**D**) in each group. (**E**). Serum OCN levels were detected by an enzyme-linked immunosorbent assay. The data are presented as the mean ± standard deviation (*n* = 5 rats per group). a, Compared with the sham group; ^*^P < 0.05; b, compared with the EP group, ^*^P < 0.05; c, compared with the Cur gel group, ^*^P < 0.05; d, compared with the ZNP gel group, ^*^P < 0.05. Cur, curcumin; ZNP, zinc oxide nanoparticle; EP, experimental periodontitis; OCN, osteocalcin; OPG, osteoprotegerin

Furthermore, Cur/ZNP gel treatment increased the expression of OCN and OPG (Fig. 5A-E). The OCN expression in the Cur/ZNP gel group was also greater than that in the ZNP gel group (Fig. 5A and C). However, the effect of the Cur/ZNP gel on the serum OCN concentration was not significantly different from that of Cur or ZNP (Fig. 5E). Moreover, there was no significant difference in the number of OPG-positive cells between the Cur/ZNP gel group and the Cur gel or ZNP gel groups (Fig. 5B and D).

## Discussion

The application of local drugs is an important adjuvant method for periodontal therapy. Local drug delivery systems (LDDSs) have obvious advantages in clinical applications. For instance, LDDSs require a small amount of drug and can deliver the drug in situ in periodontal pockets [25]. Cur has anti-inflammatory and antioxidant effects and could be used as a local drug for periodontal therapy. In the present study, we synthesized a slow-release Cur/ZNP hydrogel and found that it has a better antibacterial effect and is better at alleviating periodontal bone resorption in experimental periodontitis rats than Cur hydrogel alone. This study is the first to report the synergistic effect of Cur hydrogels and ZNP hydrogels in the treatment of periodontitis.

The in vitro data showed that the Cur hydrogel effectively suppressed the growth and EPS production of *P. gingivalis*. Interestingly, the Cur/ZNP hydrogel had synergistic inhibitory effects on *P. gingivalis*. This was consistent with the findings of previous studies, which demonstrated that Cur/ZNPs had a lower minimum inhibitory concentration and minimum bactericidal concentration for *Staphylococcus aureus* and *Escherichia coli* [26, 27]. Cur has been reported to have antibacterial, antiviral, and antifungal effects [28]; moreover, Cur can significantly inhibit the growth of a variety of periodontal pathogens, including *P. gingivalis*, *Prevotella intermedia*, *F. nucleatum*, and *Treponema denticola* but has no inhibitory effect on *Aggregatibacter actinomycetemcomitans* [29]. Thus, whether the Cur/ZNP hydrogel has synergistic effects on other periodontal microbiota needs to be studied further. The good biocompatibility of the Cur/ZNP hydrogel was confirmed by in vitro cytotoxicity studies in which HOK-1 cells were used; these findings are consistent with those of previous studies in which fibroblasts, MCF-7 cells and MC3T3-E1 cells were used [30, 31].

The functional role of the Cur/ZNP hydrogel in periodontal destruction was also confirmed in a model of experimental periodontitis. Various etiologies and risk factors are involved in the development of periodontitis, among which the most important are the oral microbiota and the host immune inflammatory response [32]. Notably, a comparison of the transcriptional landscape of the gingiva from rats revealed that DEGs between the sham and EP groups were significantly enriched in the immune effector process GO terms. The Cur/ZNP hydrogel notably induced the expression of genes involved in regulating bone resorption. Therefore, we investigated the effect of the Cur/ZNP hydrogel on alveolar bone loss in rats. The data indicated that both the Cur hydrogel and the ZNP hydrogel alleviated bone destruction in rats. Moreover, evidence indicates that Cur can alleviate periodontitis. For instance, local application of 2% Cur gel to the periodontal area of rats could reduce the gingival index (GI) and PD [33]. SRP assisted with 2% Cur gel had a lower PD and BI compared with SRP alone in patients with periodontitis, and it significantly reduced the PLI, BI, and PD compared with 0.2% chlorhexidine gel-assisted SRP [8, 34]. However, the osteoprotective effects of Cur remain controversial. Consistent with our findings, Cur has been shown to decrease the number of osteoclasts and inhibit alveolar bone absorption [6, 35]. Conversely, some studies have shown that oral administration of Cur does not improve the level of alveolar bone in experimental animals with periodontitis [36, 37]. We speculate that this difference may be due to the low bioavailability of Cur. Bioavailability may be improved by the hydrogel and the method of administration used in this study. Bioavailability can also be improved through chemical modifications. For example, chemically modified curcumin (CMC 2.24) can inhibit periodontitis-related bone resorption in experimental animals; inhibit osteoclast formation; reduce the infiltration of polynuclear cells and monocytes; decrease the degradation of periodontal collagen fibers; and decrease the levels of IL-1β, IL-6, TNF-α, and matrix metallopeptidase 9 (MMP-9) [38].

Cur/ZNPs significantly alleviated bone destruction and increased OCN and OPG levels. Moreover, Cur/ZNPs had an osteoprotective effect, which was reflected by the shorter CEJ-ABC distance and greater BV/TV. However, there was no significant difference between the effects of the Cur hydrogel and Cur/ZNP hydrogel on the expression of OCN and OPG in periodontal tissues. Compared with the ZNP hydrogel, the Cur/ZNP hydrogel had a stronger effect on OCN expression in periodontal tissues but had no such effect on OCN expression in the serum; thus, in addition to inducing the expression of OCN and OPG, the Cur/ZNP hydrogel may suppress alveolar bone resorption via other mechanisms. Thus, further studies are needed to confirm whether Cur/ZNPs have synergistic bone protection effects. Carcinoembryonic antigen-related cell adhesion molecule 1 (Ceacam1) is a member of the immunoglobulin superfamily related to inflammation, tumorigenesis, and bone remodeling [39]. The lower expression of Ceacam1 in the gingiva of rats in the Cur/ZNP gel group than in the Cur gel group suggested that the osteoprotective effect of the Cur/ZNP gel may be associated with Ceacam1. However, the protein expression of Ceacam1 in periodontitis, the role of Ceacam1 in alveolar bone remodeling, and the interaction between Ceacam1 and CD47 were not examined in the present study. Hence, the mechanisms underlying why the Cur/ZNP gel can better suppress alveolar bone resorption than the Cur gel require further investigation. The present study confirmed the antibacterial activity and protective effect of Cur/ZNPs on alveolar bone resorption in hydrogels, suggesting that these materials are suitable candidates for biomedical applications. However, its effect on other microorganisms in subgingival plaques needs further study. Furthermore, a more comprehensive characterization of the formulation in terms of stability and even clinical studies are needed.

## Conclusions

Cur and ZNPs had synergistic inhibitory effects on *P. gingivalis* and good biocompatibility. Moreover, the Cur/ZNP hydrogel had a stronger protective effect on alveolar bone resorption than both the Cur hydrogel and the ZNP hydrogel. In conclusion, Cur/ZNP hydrogel has great potential for use in the treatment of periodontitis, especially in inhibiting periodontal pathogenic bacteria and alleviating alveolar bone destruction.

### Electronic supplementary material

Below is the link to the electronic supplementary material.

Supplementary Material 1

## Data Availability

The data and materials used for the current study can be obtained by contacting the corresponding author.
