# Supplementary material for: Characterization and application of in situ curcumin/ZNP hydrogels for periodontitis treatment
Source: BMC Oral Health. 2024 Mar 28;24:395. doi: 10.1186/s12903-024-04054-7 (PMC10976734; doi:10.1186/s12903-024-04054-7)
Supplement: Supplementary file 1 — Supplementary Material 1 [file 12903_2024_4054_MOESM1_ESM.docx]

**Additional files**

**Characterization and application of in-situ curcumin/ZNPs hydrogel for periodontitis treatment**

**
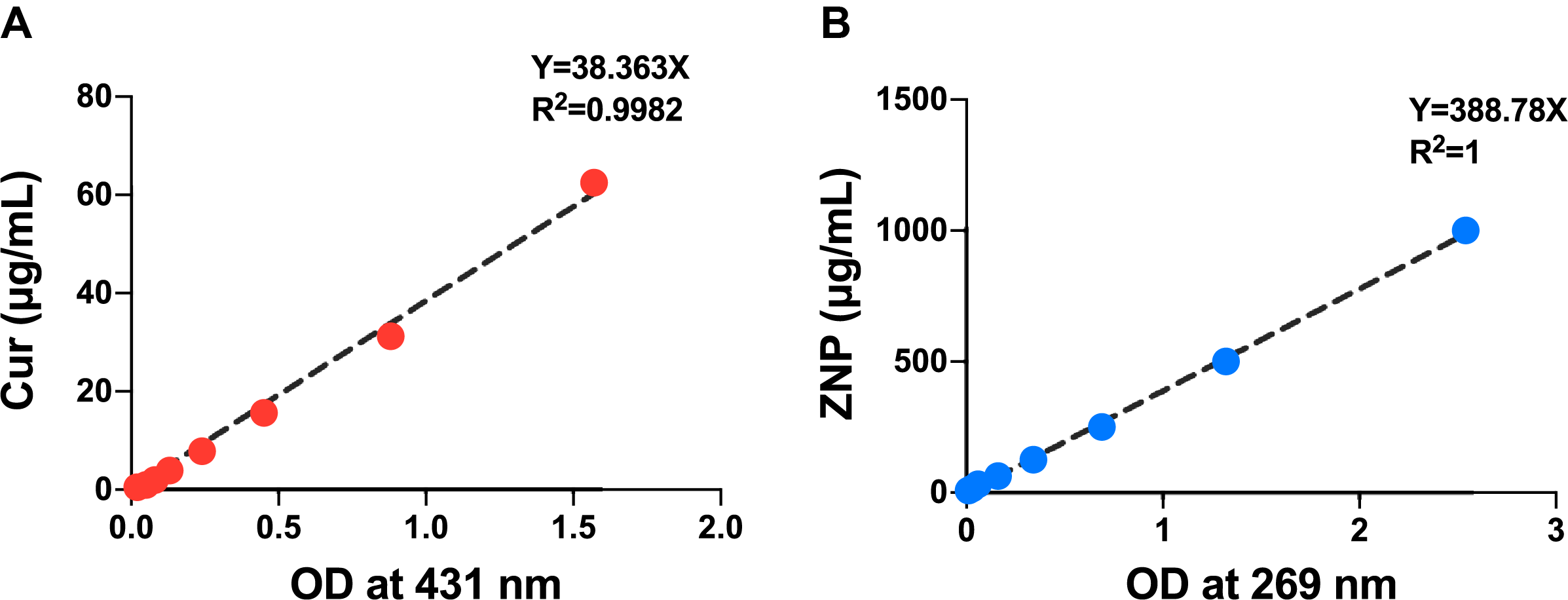
**

**Additional Figure. 1 The standard curves of Cur and ZNP.** Cur powder (2 mg) was weighed with a microelectronic balance, dissolved in 1 mL of PBS containing 0.5% Tween-80, and diluted to 62.5 μg/mL, 31.25 μg/mL, 15.63 μg/mL, 7.81 μg/mL, and 3.91 μg/mL. μg/mL, 1.95 μg/mL, 0.98 μg/mL, and 0.49 μg/mL. The optical density (OD) at 431 nm was measured with a spectrophotometer, and a standard curve of the Cur solution was prepared. The aqueous ZnO dispersion was dissolved in the same buffer medium and diluted to 1000, 500, 250, 125, 62.5, 31.25, and 15.63 μg/mL. mL, 7.81 μg/mL. The OD_268nm_ was measured, and a standard curve was constructed for the ZNP solution. Each experiment was repeated three times, and representative results are shown here.


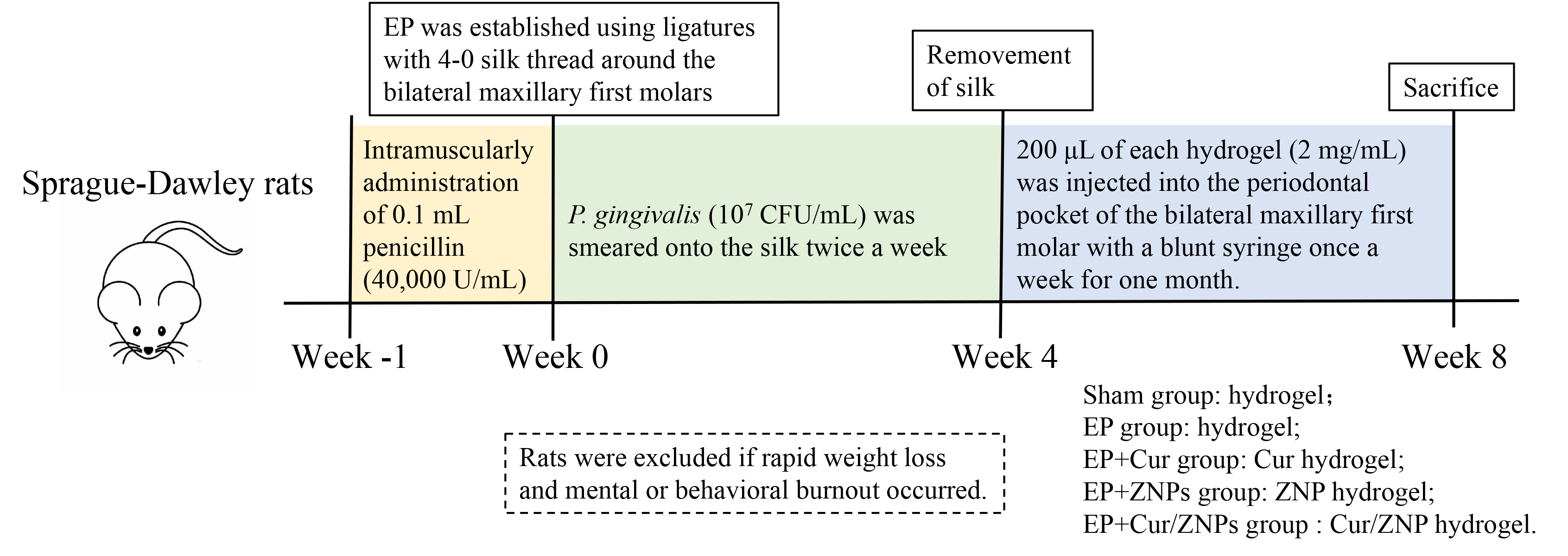


**Additional Figure. 2 The procedure of animal study.** Sprague-Dawley rats were anesthetized with isoflurane (5% induction, 2% maintenance, sealed until euthanized) and intramuscularly administered 0.1 mL penicillin (40,000 U/mL) for one week. EP was established using ligatures with 4-0 silk thread around the bilateral maxillary first molars. *P. gingivalis* ATCC 33277 (10^7^ CFU/mL) was smeared onto the silk twice a week. One month after ligature placement, rats in the EP+Cur group were treated with Cur hydrogel (Dalian Meilun Biotech Co., Ltd); the EP+ZNPs group was treated with ZNP hydrogel (Shanghai Aladdin Biochemical Technology Co., Ltd); and the EP+Cur/ZNPs group was treated with Cur/ZNP hydrogel. To do so, 200 μL of each hydrogel (2 mg/mL) was injected into the periodontal pocket of the bilateral maxillary first molar with a blunt syringe once a week for one month. Hydrogels were used as a control for the sham group. Rats were excluded if rapid weight loss and mental or behavioral burnout occurred. According to the distribution of different stages in the experiment, there were corresponding records on the label outside the cage. Outcome evaluation and data analysis were carried out based on random cage units by one investigator who was blinded to the group assignments in the experiments.


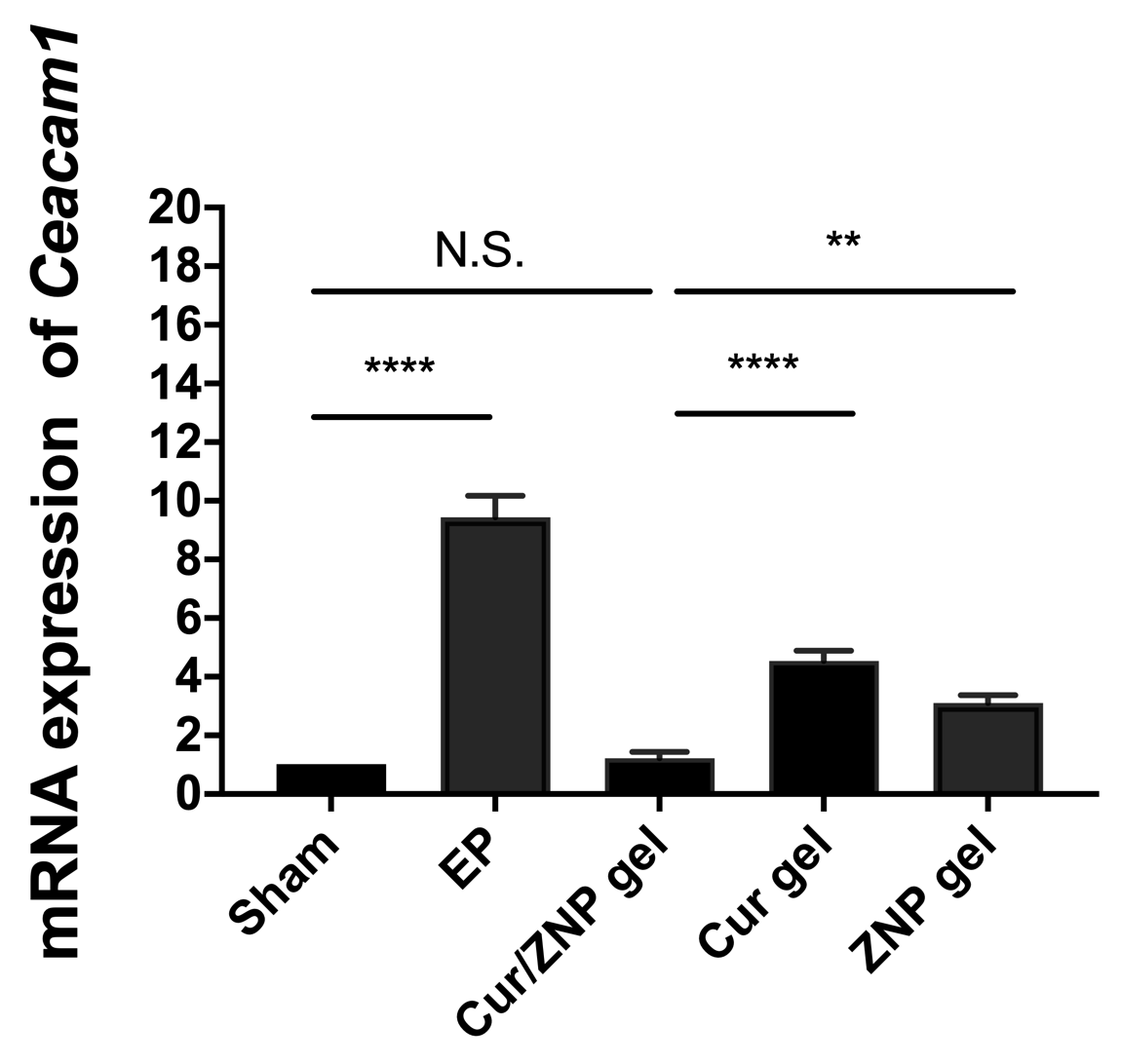


**Additional Figure. 3 Validation of *Ceacam1* expression.** The expression level of *Ceacam1* in gingiva determined by quantitative reverse transcription PCR. Data are presented as mean ± SD. ^**^P＜0.01,^****^P＜0.0001.

**Additional Table. 1. Weight of rats during the experimental period**

| Study group | Baseline (g) | Four weeks (g) | Eight weeks (g) |
| --- | --- | --- | --- |
| Sham | 287.40±7.99 | 386.40±12.95 | 425.80±7.92 |
| EP | 280.80±11.61 | 379.40±38.21 | 424.60±49.90 |
| Cur gel | 273.80±16.83 | 365.60±25.22 | 416.60±24.76 |
| ZNP gel | 283.00±7.52 | 360.20±18.78 | 403.00±20.58 |
| Cur/ZNP gel | 284.00±14.00 | 369.80±14.96 | 424.60±26.50 |

There were no significant differences in weight at baseline, before drug administration (four weeks) and at sample collection (eight weeks).

**Additional Table. 2. Injectability and fluidity of hydrogel at different temperatures**

| Temperature | 4 ℃ | 20 ℃ | 37 ℃ |
| --- | --- | --- | --- |
| Fluidity | ++ | + | - |
| Injectability | +++ | ++ | + |
